# P-2289. Gut Colonization With Multidrug-Resistant Organism And Its Impact On Bloodstream Infection In Children With Hematological Malignancies, A Prospective Observational Study

**DOI:** 10.1093/ofid/ofae631.2442

**Published:** 2025-01-29

**Authors:** Reham Khedr, Nadine Abdelwahab, Dalia Kadry, Rana Hamdy

**Affiliations:** Children's cancer hospital Egypt 57357/ National Cancer Institute - Cairo university, cairo, Al Qahirah, Egypt; National Cancer Institute - Cairo University, Cairo, Al Qahirah, Egypt; National Cancer Institute - Cairo University, Cairo, Al Qahirah, Egypt; National Cancer Institute - Cairo University, Cairo, Al Qahirah, Egypt

## Abstract

**Background:**

Preventing infections due to MDR pathogens is particularly important in the era of MDR pathogens. Once colonization with an MDR strain occurs, the risk of subsequent infection is exceptionally high in neutropenic patients. For carbapenem-resistant Enterobacteriaceae, rectal colonization by resistant pathogens was followed by BSIs in 45% of neutropenic patients. The association between colonization and subsequent infection has been reported for many MDR bacteria, such as VRE, ESBL-producing Enterobacteriaceae, P.aeruginosa, S. maltophilia, and carbapenem or colistin-resistant K. pneumoniae

**Methods:**

A prospective observational study was conducted at the National Cancer Institute on 70 children with hematological malignancies. Rectal swabs were collected at admission and weekly to screen for MDRO colonization. Demographic, clinical, and microbiological data were collected.

**Results:**

At admission, 60% of children were colonized with MDROs. The most common MDROs were Escherichia coli (66%) [with CRE comprising 60% and ESBL 40%], Klebsiella CRE (16%), and methicillin-resistant Staphylococcus aureus (MRSA) (5%). Risk factors for MDRO colonization included previous antibiotic exposure, intensive chemotherapy, and prolonged hospitalization. During the study period, (28%) of the children developed BSI. Of these, (60 %) were caused by MDROs. MDRO colonization was significantly associated with an increased risk of MDRO BSI. Also, intrabdominal infection (Typlitis ) and Perianal Infection were reported in 15% and 14% of the MDRO-colonised children.

**Conclusion:**

Gut colonization with MDROs is common in children with hematological malignancies and is associated with an increased risk of MDRO BSI. Strict infection control measures and judicious antibiotic use are crucial to prevent MDRO colonization and subsequent BSI in this vulnerable population.

**Disclosures:**

All Authors: No reported disclosures

